# Tumor-specific T cells signal tumor destruction via the lymphotoxin β receptor

**DOI:** 10.1186/1479-5876-5-14

**Published:** 2007-03-13

**Authors:** Hauke Winter, Natasja K van den Engel, Christian H Poehlein, Rudolf A Hatz, Bernard A Fox, Hong-Ming Hu

**Affiliations:** 1 Laboratory of Molecular and Tumor Immunology, Robert W. Franz Cancer Research Center, Earle A. Chiles Research Institute, Providence Portland Medical Center, Portland, Oregon, USA; 2 Department of Surgery, Klinikum Grosshadern, LMU Munich, Marchioninistr. 15, 81377, Munich, Germany; 3 Department of Molecular Microbiology and Immunology and the OHSU Cancer Institute, OHSU, Portland, Oregon, USA; 4 Laboratory of Cancer Immunobiology, Robert W. Franz Cancer Research Center, Earle A. Chiles Research Institute, Providence Portland Medical Center, Portland, Oregon, USA; 5 Department of Radiation Oncology and the OHSU Cancer Institute, OHSU, Portland, Oregon, USA

## Abstract

**Background:**

Previously, we reported that adoptively transferred perforin k/o (PKO), and IFN-γ k/o (GKO), or perforin/IFN-γ double k/o (PKO/GKO) effector T cells mediated regression of B16BL6-D5 (D5) pulmonary metastases and showed that TNF receptor signaling played a critical role in mediating tumor regression. In this report we investigated the role of lymphotoxin-α (LT-α) as a potential effector molecules of tumor-specific effector T cells.

**Methods:**

Effector T cells were generated from tumor vaccine-draining lymph node (TVDLN) of wt, GKO, LT-α deficient (LKO), or PKO/GKO mice and tested for their ability to mediate regression of D5 pulmonary metastases in the presence or absence of LT-βR-Fc fusion protein or anti-IFN-γ antibody. Chemokine production by D5 tumor cells was determined by ELISA, RT-PCR and Chemotaxis assays.

**Results:**

Stimulated effector T cells from wt, GKO, or PKO/GKO mice expressed ligands for LT-β receptor (LT-βR). D5 tumor cells were found to constitutively express the LT-βR. Administration of LT-βR-Fc fusion protein completely abrogated the therapeutic efficacy of GKO or PKO/GKO but not wt effector T cells (p < 0.05). Consistent with this observation, therapeutic efficacy of effector T cells deficient in LT-α, was greatly reduced when IFN-γ production was neutralized. While recombinant LT-α1β2 did not induce apoptosis of D5 tumor cells in vitro, it induced secretion of chemokines by D5 that promoted migration of macrophages.

**Conclusion:**

The contribution of LT-α expression by effector T cells to anti-tumor activity in vivo was not discernable when wt effector T cells were studied. However, the contribution of LT-β R signaling was identified for GKO or PKO/GKO effector T cells. Since LT-α does not directly induce killing of D5 tumor cells in vitro, but does stimulate D5 tumor cells to secrete chemokines, these data suggest a model where LT-α expression by tumor-specific effector T cells interacts via cross-linking of the LT-βR on tumor cells to induce secretion of chemokines that are chemotactic for macrophages. While the contribution of macrophages to tumor elimination in our system requires additional study, this model provides a possible explanation for the infiltration of inate effector cells that is seen coincident with tumor regression.

## Background

Adoptive transfer of tumor-specific T cells can induce tumor regression in animal models and occasionally in patients with cancer [1,2]. However, the mechanisms for T cell mediated tumor regression are still under intensive investigation. Tumor-specific T cells process multiple effector molecules that can potentially participate in various pathways leading to tumor destruction in vivo. Previously, we have documented that tumor regression mediated by adoptive transfer of tumor-specific effector T cells could be independent of either perforin or IFN-γ pathways [3,4]. Recently, we also demonstrated that effector T cells lacking both perforin and IFN-γ could mediate regression of pulmonary metastases of melanoma and fibrosarcoma, albeit the efficacy was greatly reduced [5], demonstrating that perforin/granzyme and IFN-γ-dependent mechanisms may have a compensatory role. However, the fact that tumor regression did occur in a system lacking both perforin and IFN-γ indicates that other mechanisms, such as TNF-mediated pathways, can orchestrate tumor regression [5].

IFN-γ is known to play a central role in the immune surveillance against tumors [6-8]. In several murine tumor models the therapeutic efficacy of adoptively transferred effector T cells strongly correlates with their tumor-specific IFN-γ release. Barth et al., and others observed a direct correlation between the therapeutic efficacy of tumor infiltrating lymphocytes (TIL) and their tumor-specific IFN-γ production in a murine sarcoma model [9]. Similar correlations between therapeutic efficacy and the tumor specific IFN-γ production were found for effector T cells derived from lymph nodes (LN) draining the vaccine sites of MCA-205 sarcoma or B16BL6 melanoma tumor cell lines [10-13]. We also recently showed that a T1 phenotype is crucial for their therapeutic efficacy [14]. When therapeutic effector T cells from wt TVDLN are cultured in a T2 promoting cytokine milieu with IL-4 and anti-IL-12 antibody, they lost their therapeutic efficacy. So far, two major classes of effector molecules that have been identified. First, effector molecules are able to mediate the direct killing of tumor targets – perforin and granzymes in the granules of CTL and ligands for death receptors on the cell surface of T cells. Second, IFN-γ produced by tumor-specific T cells mediates tumor regression probably via the activation of host macrophages [9,15].

While these studies indicate that IFN-γ plays a critical role in the development of tumor immunity, we and others have recently shown, that IFN-γ is not essential for the priming of tumor specific effector cells in TVDLN or as an effector molecule of adoptively transferred T-cells [4,15,16]. This observation led to the hypothesis that other T1 cytokines might play an essential role for the therapeutic efficacy of tumor-specific effector T cells and might compensate for the loss of IFN-γ in GKO mice.

Because no evidence for the generation of type 2 cytokine T cell immune responses was observed in GKO mice, we hypothesized that other type 1 cytokines produced by adoptively transferred T cells were critical for the therapeutic efficacy. LT-βR ligand, a membrane bound heterotrimer known as LT-α1β2, was found to be expressed abundantly on recently activated Th1 T cells [17-19]. In addition, a recently described ligand for LT-βR (LIGHT) was found to be expressed on activated lymphocytes and shown to be able to induce secretion of chemokines and apoptosis of some tumor cell lines [20-24]. Meanwhile, LT-βR was found to be expressed on non-lymphoid cells and the majority of tumor cell lines [18,25-27]. To investigate whether ligands for LT-βR, LT-α1β2 (and/or LIGHT), could be the effector molecules of effector T cells adoptive transfer experiments were designed. These studies examined how the presence or absence of IFN-γ or IFN-γ and perforin affected the contribution of LTα to T cell mediated-tumor regression. Effector T cells were generated from TVDLN of wt, GKO and adoptively transferred into wt or GKO mice with established 3 day pulmonary metastases of D5 tumor cells [5]. Signaling through LT-βR was blocked by administration of LT-βR Fc after adoptive transfer of T cells. Effector T cells deficient of membrane bound lymphotoxin LT-α1β2 were also generated from TVDLN by vaccinating RAG1 mice reconstituted with naïve spleen cells from LKO mice. The therapeutic efficacy of LKO effector T cells in an adoptive immunotherapy model was compared in the presence or absence of IFN-γ neutralizing antibody. To delineate a potential role of LT-βR signaling in T cell mediated tumor regression, recombinant LT-α1β2 was used for the further investigation of the effect of LT-βR signaling on D5 tumor cells in vitro.

## Materials and methods

### Mice

Female C57BL/6J (wt), GKO (C57BL/6-IFN-γ ^tm1Ts^), and LKO (C57BL/6 -LT^tm1Sdz^) mice were purchased from the Jackson Laboratory (Bar Harbor, ME) and maintained in a specific pathogen-free environment. Perforin and IFN-γ double deficient (PKO/GKO) mice were generated as described previously (5). Mice were generally 8 to 12 weeks old at the time of experimentation. Recognized principles of laboratory animal care were followed (Guide for the Care and Use of Laboratory Animals, National Research Council, 1996), and all animal protocols were approved by the Earle A. Chiles Research Institute Animal Care and Use Committee.

### Tumor cell lines

D5 is a poorly immunogenic subclone of the spontaneously arising B16BL6 melanoma [10] (provided by Dr. S. Shu, Cleveland Clinic Foundation, Cleveland, OH). An early passage of the original BL6 tumor was provided by Dr. E. Gorelick and was subcloned by limiting dilution culture in Dr. S. Shu's laboratory. D5 exhibits low to undetectable class I (H-2 D^b ^and K^b^) expression and no class II expression. D5-G6 is a stable clone of D5 that was originally transduced with a murine GM-CSF retroviral MFG vector (provided by Dr. M. Arca, University of Michigan, Ann Arbor, MI) [44]. D5-G6 cells secrete approximately 200 ng/ml/10^6 ^cells/24 h GM-CSF.

### Culture conditions

Lymphocytes and tumor cells were cultured in complete medium (CM), which consisted of RPMI 1640 containing 0.1 mM nonessential amino acids, 1 mM sodium pyruvate, 2 mM L-glutamine, and 50 μg/ml of gentamicin sulfate (Bio Whittaker, Walkersville, MD.). This was further supplemented with 50 mM 2-mercaptoethanol (Aldrich, Milwaukee, WI, USA.), and 10% fetal bovine serum (GIBCO BRL, Grand Island, NY). Tumor cells were harvested 2-to 3 times per week by brief trypsinization and maintained in T-75 or T-150 culture flasks.

### Generation of effector T cells from TVDLN

D5-G6 tumor cells were harvested by trypsinization, washed twice with HBSS and resuspended at 2 × 10^7 ^cells per ml. One million D5-G6 tumor cells were injected s.c. into both hind and fore flanks of wt, or GKO mice. Eight days following vaccination, the draining superficial inguinal and auxiliary lymph nodes were harvested. TVDLN were resuspended at 2 × 10^6 ^cells per ml in CM and cultured in 24 well plates with 50 μl of a 1:40 dilution of 2c11 ascites (anti-CD3) as described previously [3]. After two days of activation the T cells were harvested and expanded in CM containing 60 IU rhIL-2/ml for three additional days. T cells were then harvested, washed twice in HBSS, counted and used in adoptive transfer and cytokine release assays.

### Adoptive immunotherapy

Experimental pulmonary metastases were established by i.v. inoculation of 2 × 10^5 ^D5 tumor cells. Three days later effector T cells were adoptively transferred i.v. via tail vein. Starting on the day of T-cell infusion, mice received 90,000 IU recombinant human IL-2 (provided by Chiron, Emeryville, CA) i.p. once per day for four days. Animals were sacrificed 11 to 13 days following tumor inoculation by CO2 narcosis and their lungs were harvested and fixed in Fekete's solution. Where indicated, neutralizing LT-βR Fc or control human IgG were administered i.v. before the adoptive transfer of T cells and for the following three days. The number of pulmonary metastases was counted in a blinded fashion. Metastases that were too numerous to count accurately were known to be greater than 250 metastases and were assigned a value of 250.

### Statistical analysis

The statistical significance of differences in the number of metastases between experimental groups was determined by the Wilcoxon rank sum test. Two-sided p values of < 0.05 were considered significant. Each treatment group consisted of at least 5 mice, and no animal was excluded from the statistical evaluations.

### Apoptosis induction

D5 tumor cells were incubated with different concentration of recombinant mouse LT-α1β2 (Sigma, MO) with or without cycloheximide (CHX) (10 μg/ml) in 500 μl CM in 24 well plates. 24 hours later the cells were harvested, washed twice with ice cold HBSS and resuspended in 100 μl Annexin binding buffer. Apoptosis was determined by staining with Annexin-V-FITC (Pharmingen) and counterstaining with 10 μl propidium (50 g/ml in PBS). 15 minutes later, the cells were analyzed by FACS and the amount of apoptotic cells determined by calculating the percentage of cells staining positive with Annexin-V.

### RT-PCR

D5 cells were cultured in CM alone, with an indicated number of effector T cells generated as above, or with an indicated concentration of LT-α1β2. After 4–24 hours incubation, the total RNA was extracted from D5 cells, or after removal of T cells by washing three time with PBS, using the Qiagen Rneasy mini kit (Qiagen, CA). 2 μg of RNA was denatured and reversely transcribed to cDNA using the oligo dT (15) primer (Roche) and MMLV reverse transciptase (Invitrogen, CA). Thermocycling conditions were: denaturing at 94°C for 30', annealing at 55°C for 30', and extending at 72°C for 30'. A total of 25 cycles were performed. The DNA sequences of primers used are shown in Table 1.

**Table 1:** Primer sequences used for RT-PCR

***Gene***	***Forward primer sequence ***	***Reverse primer sequence***
MIP-1α	5'-atg aag gtc tcc acc act gcc ctt g-3'	5'-ggc att cag ttc cag gtc agt gat-3'
MIP-1β	5'-gtt ctc agc acc aat ggg ctc tga-3'	5'-ctc tcc tga agt ggc tcc tcc tg-3'
IP-10	5'-cct atc ctg ccc acg tgt tg-3'	5'-cgc acc tcc aca tag ctt aca-3'
RANTES	5'-cat cct cac tgc agc cgc c-3'	5'-cca agc tgg cta gga cta gag-3'
MIG	5'-atg aag tcc gct gtt ctt ttc-3'	5'-tta tgt agt ctt cct tga acg ac-3'
HPRT	5'-gtt gga tac agg cca gac ttt gtt g-3'	5'-gag ggt agg atg gcc tat agg ct-3'

### Measurement of cytokines

After activation and expansion TVDLN were washed, resuspended in CM, supplemented with IL-2 (60 IU/ml) and seeded at 4 × 10^6^/2 ml/well in a 24 well plate. The cells were either cultured without further stimulation or stimulated with 2 × 10^5 ^D5, MCA-310 tumor cells, or immobilized anti-CD3 (positive control). Supernatants were harvested after 24 hours and assayed for the release of KC and RANTES by ELISA using commercially available reagents (Pharmingen). The concentration of cytokines in the supernatant was determined by regression analysis.

### Chemotactic assay

D5 tumors cells (10^5 ^well) were plated in the bottom chamber of a 24 well transwell plate (Corning Costar, Cambridge, MA) in CM. Two hours later they were stimulated with or without LT-α1β2 (100 ng/ml). After 12 hours 3.5 × 10^5 ^DJ2PM macrophage cells were resuspended in 250 μl CM and plated into the upper chamber of a transwell plate (5 μm pore size). After 4 h the cells in the bottom chamber were trypsinized, harvested, and washed 2 × in PBS and stained with anti-CD11b antibody (Pharmingen). The number of macrophages that migrated into the lower well was determined by FACS analysis as the percentage of CD11b positively stained cells.

## Results

### D5 tumor cells express LT-βR while effector T cells express the ligands

**Figure 1:**
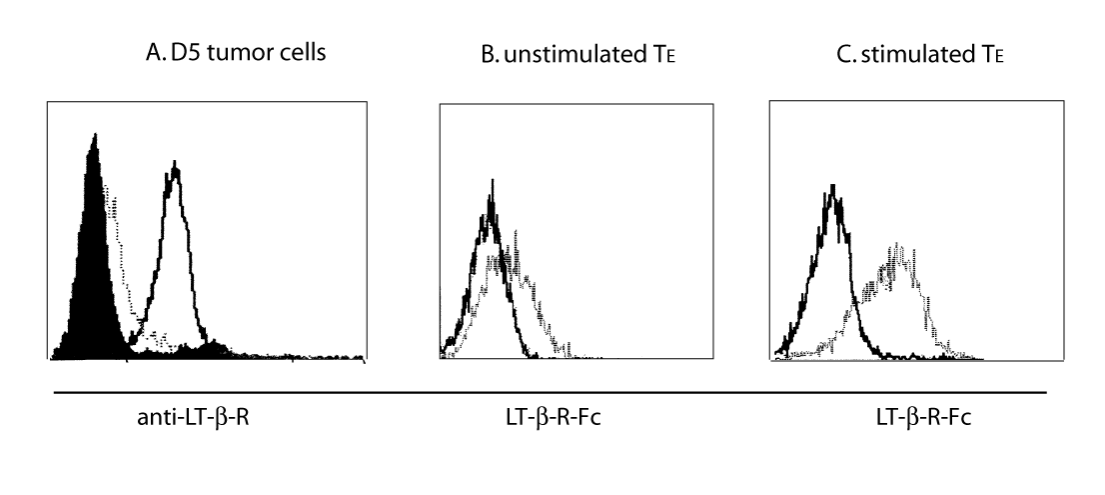
**Expression of LT-βR and its ligands**. (A) D5 tumor cells were first stained with monoclonal rat anti-mouse LT-βR antibody (kind gift from Dr. M. Croft, La Jolla Institute for Allergy and Immunology) and isotype control antibody, and then with FITC-labeled goat anti-rat IgG (Jackson immune research laboratory). After staining, cells were analyzed by flow cytometry. D5 alone, filled histogram; isotype, dashed line; anti-mouse LT-βR, solid line. Effector T cells that were not stimulated (B) or stimulated with anti-CD3 and CD28 antibodies for 6 hours (C) were incubated with mouse LT-βR-Fc fusion protein or control Fc fusion protein (509-Fc), and stained with PE-labeled goat anti-human Fc antibody. Stained cells were analyzed by flow cytometry. Solid line, control Fc fusion protein; dashed line, LT-βR Fc fusion protein.

LT-βR expression was mainly found on non-lymphoid tissues and tumor cell lines [18]. In contrast, the expression of the ligands for LT-βR (LT-α1β2 or LIGHT) is highly restricted to activated lymphocytes [18,21]. First, the D5 melanoma cell line used for the majority of our studies was found to express a high level of LT-βR (Figure 1A). Next, the expression of its cognate ligand on either wt or GKO effector T cells generated from TVDLN was examined by staining with LT-βR-Fc-fusion protein and PE-conjugated anti-human Fc antibody. Both wt and GKO TE expressed a low but detectable level of binding to LT-βR-Fc compared to control Fc fusion protein (509-Fc) (Figure 1B). After stimulation with anti-CD3 and CD28 antibodies for 6 hours, a marked increase in binding of LT-βR-Fc on both GKO and wt effector cells was observed. No difference of binding was found between wt and GKO effector T cells before or after in vitro activation with anti-CD3 and CD28 antibodies.

### Blocking the therapeutic efficacy of GKO, but not wt effector T cells, by LT-βR-Fc

Because D5 tumor cells expressed the LT-βR, while wt and GKO effector T cells expressed the cognate ligands for LT-βR, a possible role of LT-βR signaling in tumor regression after adoptive transfer was investigated in an experimental pulmonary metastasis model. The LT-βR-Fc fusion protein was administered i.v. before and after the adoptive transfer of wt and GKO effector T cells into wt or GKO mice bearing 3day established D5 pulmonary metastases. As shown in table 2, blocking the LT-βR signaling did not affect the therapeutic efficacy of wt effector T cells in 2 of 2 experiments performed, while the antitumor activity of GKO effector T cells was abrogated in 3 of 4 consecutive experiments. These experiments suggested a significant role of LT-βR signaling for the tumor regression in certain circumstances of tumor rejection if effector T cells failed to produce IFN-γ.

**Table 2:** The effect of LT-βR-Fc fusion protein administration on adoptive immunotherapy.

***Adoptive immunotherapy^a^***				***Mean number of pulmonary metastases^b^***			
**Donor**	**Hosts**	**Number of T cells transferred**	**Blocking proteins^b^**	**Exp.1**	**Exp.2**	**Exp.3**	**Exp.4**

None	wt	0	none	250	250		
wt	wt	35	hu IgG	21(5)^d^	52(13)^d^		
wt	wt	35	LTβR-Fc	33(6)^d^	78(11)^d^		
wt	GKO	0	None	243(60)	244(60)	250	250
GKO	GKO	35	hu IgG	6(3)^d^	88(23)^d^	85(12)^d^	90(30)^d^
GKO	GKO	35	LTβR-Fc	9(3)^d^	211(56)^*e*^	250^e^	250^e^

a) Mice were vaccinated s.c. with D5-G6 tumor cells and TVDLN were harvested 8 days later. Lymph node cells were stimulated in vitro with anti-CD3 for two days and then expanded for three days in 60 IU/ml IL-2. Effector cells were harvested and 35 × 10^6 ^T cells were adoptively transferred into animals with established 3-day D5 pulmonary metastases. IL-2 (90,000 IU) was administered daily i.p. for four consecutive days following adoptive transfer.

b) Purified control human IgG or LT-βR-Fc (250 μg) was directly administered i.v. after adoptive transfer of the TE and for the following 3 days once per day.

c) Mice were sacrificed 13 days following i.v. inoculation of tumor and the number of pulmonary metastases enumerated in a blinded fashion. Results presented are the mean of 5 mice. Metastases that were too numerous to count accurately were known to be greater than 250 metastases and were assigned a value of 250.

d) p < 0.05 compared to IL-2 alone treated controls.

e) p > 0.05 compared to IL-2 alone treated controls.

### IFN-γ neutralization blocked the therapeutic efficacy of LKO effector T cells

To further support the compensatory role of LT-βR and IFN-γ, LKO effector T cells were generated from RAG1 mice reconstituted with naïve spleen cells from LT-α k/o mice. Because LT-α k/o mice lack LN, it necessitates the reconstitution of RAG1 mice for the generation of TVDLN. As a control wt effector T cells were also generated from RAG1 mice reconstituted with naïve wt spleen cells. In the first experiment, both wt and LKO effector T cells were able to mediate a complete tumor regression; the administration of anti-IFN-γ antibody significantly reduced the efficacy of LKO effector T cells. In the second experiment LKO effector T cells were less effective compared to wt T cells. The administration of anti-IFN-γ antibody totally abrogated the anti-tumor activity of LKO effector T cells (Table 3). Blocking experiments with anti-IFN-γ antibody were not done for wt effector T cells in this report, since we have previously documented that the administration of anti-IFN-γ antibody did not affect therapeutic efficacy of wt effector T cells [4].

**Table 3:** The effect of IFN-γ neutralization on adoptive immunotherapy.

***Adoptive immunotherapy^a^***				***Mean number of pulmonary metastases^b^***	
**Donor**	**Hosts**	**Number of T cells transferred**	**Blocking proteins^b^**	**Exp.1**	**Exp.2**

None	wt	0	none	250	250
LKO	wt	15	rat IgG	0 ^d^	81(35)^d^
LKO	wt	15	Anti-IFN-γ	39(29)^d^	242(8)^e^

a)Rag1k/o mice were reconstituted with naïve 20 × 10^6 ^naïve spleen cells from naïve wt or LT-α k/o mice. They were then vaccinated s.c. with D5-G6 tumor cells, and TVDLN were harvested 8 days later. Lymph node cells were stimulated in vitro with anti-CD3 for two days and then expanded for three days in 60 IU/ml IL-2. Effector cells were harvested and 15 × 10^6 ^T cells were adoptively transferred into animals with established 3-day D5 pulmonary metastases. IL-2 (90,000 IU) was administered daily i.p. for four consecutive days following adoptive transfer.

b)Purified control rat IgG or rat anti-mouse IFN-γ antibody (250 μg) was directly administered i.v. after adoptive transfer of the T cells and for the following 3 days once per day.

c) Mice were sacrificed 13 days following i.v. inoculation of tumor and the number of pulmonary metastases enumerated in a blinded fashion. Results presented are the mean and SE of 5 mice. Metastases that were too numerous to count accurately were known to be greater than 250 metastases and were assigned a value of 250.

d) p < 0.05 compared to IL-2 alone treated controls.

e) p > 0.05 compared to IL-2 alone treated controls.

### Blocking the therapeutic efficacy of PKO/GKO effector T cells by LT-βR-Fc

The above experiments suggested that LT-βR signaling mediated by effector T cells contributed to tumor regression if IFN-γ was neutralized while the perforin-mediated cytotoxicity was intact. Our previously published data demonstrated that effector T cells from perforin and IFN-γ double deficient (PKO/GKO) mice could still mediate tumor regression in the adoptive immunotherapy model (5). We investigated whether LT-βR signaling could contribute to the tumor regression if PKO/GKO effector T cells were used. As expected, data in Table 4 showed that LT-βR-Fc could not block the function of wt effector T cells, however, it significantly diminished the therapeutic effacy of PKO/GKO effector T cells.

**Table 4:** The effect of LT-βR-Fc fusion protein administration on adoptive immunotherapy.

***Adoptive immunotherapy^a^***				***Mean number of pulmonary metastases^b^***
**Donor T cells**	**Hosts**	**Number of T cells transferred**	**Blocking proteins^b^**	

wt	wt	0	none	192(28)
wt	wt	35	none	0(0)
wt	wt	35	hu IgG	0(0)
wt	wt	35	LTβR-Fc	0(0)
PKO/GKO	wt	70	None	0(0)
PKO/GKO	wt	70	hu IgG	0(0)
PKO/GKO	wt	70	LTβR-Fc	78(50) ^d^

a) Wild type (wt) or perforin and IFN-γ double deficient (PKO/GKO) mice were vaccinated s.c. with D5-G6 tumor cells and TVDLN were harvested 8 days later. Lymph node cells were stimulated in vitro with anti-CD3 for two days and then expanded for three days in 60 IU/ml IL-2. Effector cells were harvested and 35 × 10^6 ^T cells were adoptively transferred into animals with established 3-day D5 pulmonary metastases. IL-2 (90,000 IU) was administered daily i.p. for four consecutive days following adoptive transfer.

b) Purified control human IgG or LT-βR-Fc (250 μg) was directly administered i.v. after adoptive transfer of the TE and for the following 3 days once per day.

c) Mice were sacrificed 13 days following i.v. inoculation of tumor and the number of pulmonary metastases enumerated in a blinded fashion. Results presented are the mean of 5 mice.

d) p < 0.05 compared to IL-2 alone treated controls.

### LT-α1β2 failed to induce apoptosis of D5 tumor cells

**Figure 2:**
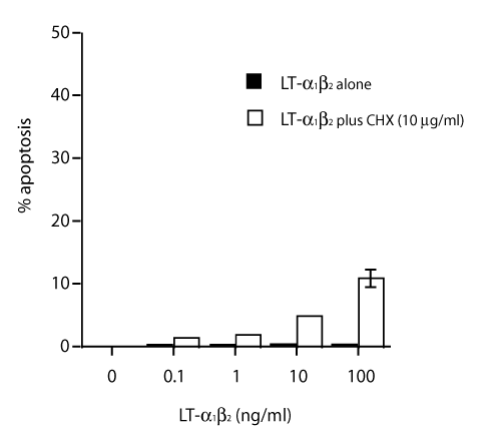
**LT-α1β2 failed to induce apoptosis of D5 tumor cells**. D5 tumor cells were incubated with indicated amount of murine recombinant LT-α1β2 with or without protein synthesis inhibitor cycloheximide (10 μg/ml) for 24 hours. Cells were then stained with Annexin-FITC. The percentage of cells that underwent apoptosis was determined by FACS analysis.

LT-α1β2 can induce apoptosis directly in some adenocarcinoma cell lines and growth arrest in melanoma cells [23,24]. One potential mechanism for LT-βR in T-cell mediated tumor regression in our model is the direct induction of apoptosis of D5 tumor cells. However, when D5 tumor cells were incubated with different doses of LT-α1β2 (1–100 ng/ml) with or without IFN-γ (200 U/ml) for 24 hours, no direct cytotoxic effect was observed (Data not shown). A low but detectable level of apoptosis (12% at 100 ng/ml of LT-α1β2) was detected in tumor cells incubated with LT-α1β2 in the presence of cycloheximide after 24 hours of incubation (Figure 2). Since significant difference in signaling and function was observed for TNF family members, our data do not preclude the possibility that membrane anchored LT-α1β2 on effector T cells could still kill tumor cells directly. These data indicate that a direct cytotoxicity of LT-βR signaling does not play a significant role in our model. Therefore an indirect pathway may provide a better explanation.

### D5 tumor cells produce chemokines and induce chemotaxis of macrophages after incubation with effector T cells or treatment with LT-α1β2

**Figure 3:**
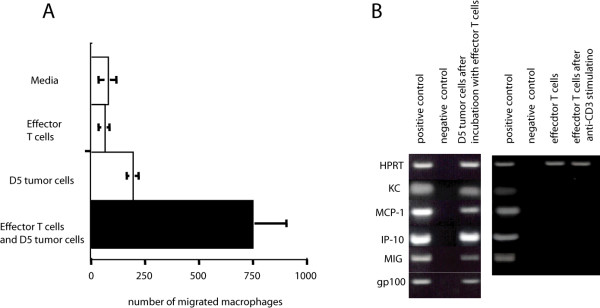
**Expression of Chemokine by D5 melanoma cells and Chemotaxis of Macrophages In Vitro**. (A) Macrophage chemotactic activity of D5 supernatant after incubation with effector T cells. Supernatant derived from cultured D5 tumor cells or D5 tumor cells that were cocultured with effector T cells for 24 hours were plated in the lower chamber of a transwell plate. The number of DJ2P macrophage cells placed on the upper chambers that trans-migrated into the lower chamber was determined by FACS analysis with FITC-labeled antiCD11b antibody. (B) Chemokine expression by D5 tumor cells or effector T cells with and without stimulation. Expression of chemokines by D5 tumor cells after cocluture with effector T cells and removal of T 24 hours late or effector T cells stimulated with anti-CD3 antibody for 24 hours were analyzed by RT-PCR. HPRT expression was used for the control of total mRNA.

Previously, we have shown that adoptive transfer of wt and GKO TE induced an influx of macrophages and granulocytes into the lungs of mice with established pulmonary metastases [4]. To examine whether the macrophage chemotaxis is induced after coculture of effector T cells and D5 tumor cells, an in vitro chemotaxis assay was used. As shown in Figure 3a, supernatant from cultured D5 melanoma cells but not unstimulated effector T cells exhibited macrophage chemotaxis activity. The number of migrated macrophages was dramatically increased when supernatant was collected from a co-culture of D5 melanoma and effector T cells. Next, we also examined the expression of chemokines inducing macrophage chemotaxis (KC, MCP-1, IP-10, and MIG) by either D5 melanoma cells or effector T cells after co-culturing. While effector T cells did not express detectable KC, MCP-1, IP-10 or MIG even after stimulation with anti-CD3 antibody, surprisingly, they were expressed by D5 melanoma cells after incubation with T cells (Figure 3b). D5 melanoma cells cultured alone failed to express these ckemokines (data not shown). This observation clearly supported our earlier observation in vivo and potential contribution of macrophages in tumor regression induced by adoptively transferred T cells [4].

**Figure 4:**
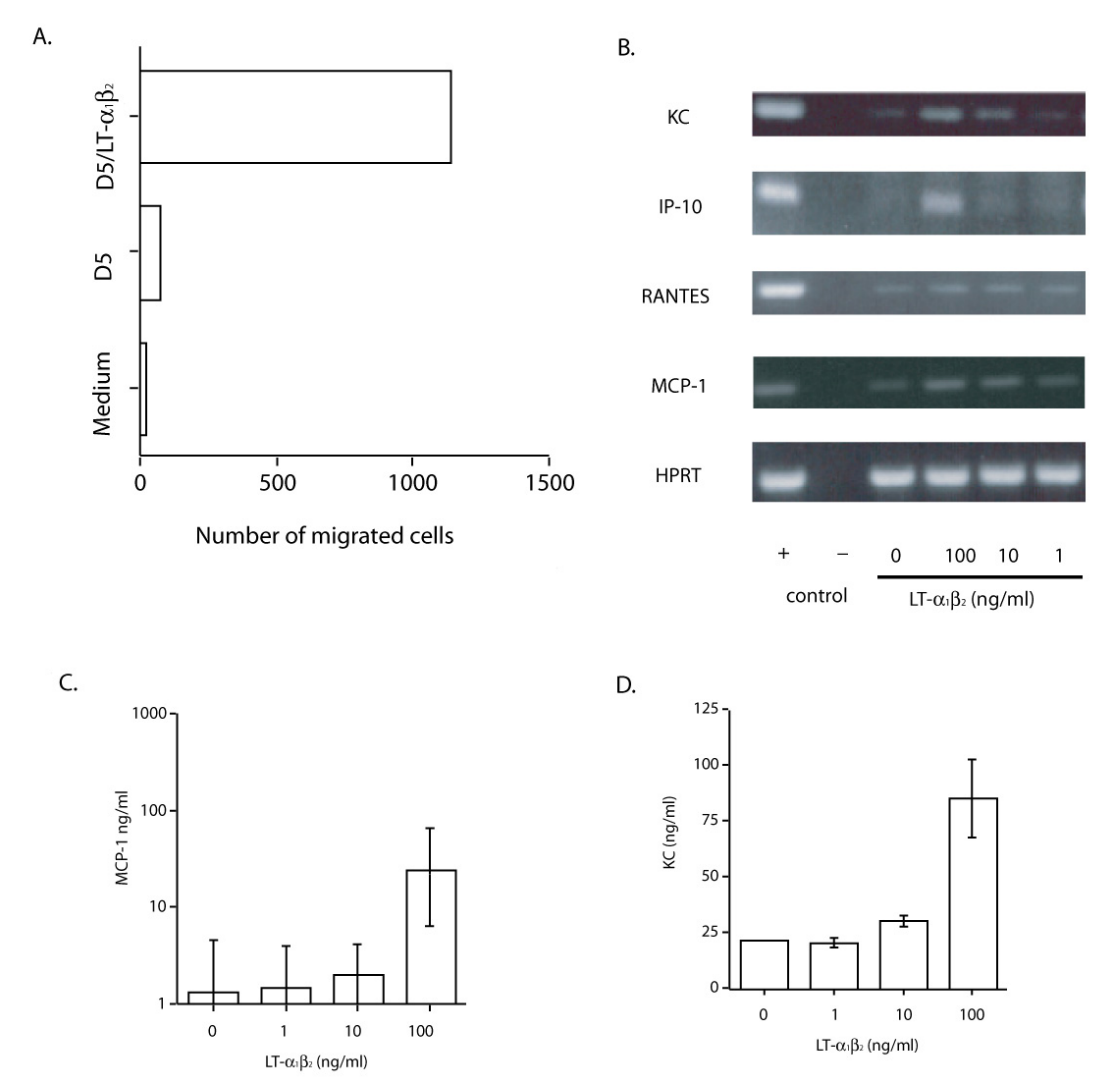
**Effect of recombinant LT-α1β2 treatment on D5 tumor cells**. (A) Macrophage chemotactic activity of D5 supernatant after treatment with LT-α1β2. Supernatant derived from cultured D5 tumor cells or D5 tumor cells that were treated with recombinant LT-α1β2 were plated in the lower chamber of a transwell plate. The number of DJ2P macrophage cells placed on the upper chambers that trans-migrated into the lower chamber was determined by FACS analysis with FITC-labeled antiCD11b antibody. (B) Chemokine expression by D5 tumor cells induced by LT-α1β2. Expression of chemokines by D5 tumor cells before or after LT-α1β2 treatment were analyzed by RT-PCR. HPRT expression was used for the control of total mRNA. Production of MCP-1 (C) and KC (D) and by D5 tumor cells in the supernatant after LT-α1β2 treatment was measured by ELISA. The error bars represents standard error of 2–3 experiments.

Degli-Esposti et al. have recently shown that activation of the LT-βR induced the production of IL-8 and RANTES in human A375 melanoma cells, indicating a possible regulatory role of LT-βR signaling in the recruitment of innate anti tumor cells such as tumoricidal macrophages [28]. We hypothesized that one possible function of LT-βR signaling in D5 tumor cells is to release chemokines that induce chemotaxis of host macrophages. Therefore D5 cells were incubated with recombinant LT-α1β2 and resultant condition media were collected. Using an in vitro chemotactic assay, the condition media from D5 tumor cells after LT-α1β2 treatment, but not untreated condition media were found to be able to attract the migration of a macrophage cell line, DJ2P (Figure 4A).

Next, the expression of the chemokines, RANTES, KC, MIP-1α, MIP-1β and MCP-1, by D5 tumor cells after LT-α1β2 treatment was examined by RT-PCR. As shown in Figure 3B, LT-α1β2 induced the expression of KC, IP-10, RANTES, and MCP-1 in D5 tumor cells, but not the expression of Mig, MIP-1α, and MIP1-β (data not shown). The levels of KC and MCP-1 proteins in treated D5 supernatant were also measured by ELISA (Figure 4C and 4D). The highest level of mRNA and proteins were observed if D5 cells were treated with the highest dose of LT-α1β2 used (100 ng/ml). Taken together, we envision that D5 tumor cells could induce the expression of membrane-bound LT-α1β2 or LIHGT on adoptively transferred effector T cells, in turn, LT-α1β2 triggered the release of multiple chemokines from D5 tumor cells and resulted in the influx of macrophages into the tumor sites.

## Discussion and conclusion

Previously, we have documented that granzyme, IFN-γ, and TNF are three primary effector mechanisms by which effector T cells could mediate tumor regression in adoptive transfer models [3-5]. The contribution by TNF family members expressed by effector T cells is more difficult to measure and less well appreciated. Our previous publication indicated that TNF could mediate tumor regression if effector T cells were deficient of both perforin and IFN-γ [15]. However, the blocking experiments with TNFR-Fc fusion could not completely abrogate the tumor regression mediated by the adoptive transfer of perforin and IFN-γ double deficient cells. Thus, other effector molecules expressed by effector T cells could play a role even if all three major effector molecules were absent or blocked. In our present study we identified that LT-βR signaling pathways also played a significant role if IFN-γ was absent in the system. One possible mechanism for LT-βR signaling is to stimulate chemokine secretion by D5 tumor cells and induce macrophage recruitment.

Cross linking of LT-βR on tumor cells by membrane bound ligands expressed on effector T cells after tumor stimulation contributed to tumor regression. In vitro experiments suggested a possible mechanism involving the recruitment of macrophages rather than a direct killing mechanism by LT-α1β2. According to this notion, Plautz et al. demonstrated that host macrophages are important for the cross-presentation of tumor antigens to adoptively transferred effector T cells during the phase of tumor eradication [16]. A critical role of LT-βR has also been demonstrated in the infectious, autoimmune diseases and transplantation rejection models [29-32]. Lucas et al. demonstrated that both TNFR and LT-βR pathways played important roles in protective immunity against *Mycobacterium bovis *BCG infection and LT-βR signaling is critical for the development of Th1 immune response, iNOS activation of macrophage, and granuloma formation [33]. LT-βR was used to reverse autoimmune diseases in various models [29,32] and to prevent transplant rejection [31]. Our results added another important function of LT-βR as an important tumor regression mechanism independent of IFN-γ.

Because LT-βR-Fc can block LT-α1β2 and LIGHT, another ligand of the TNF superfamily expressed on activated T-cells and immature DC [21,34], both LT-α1β2 and LIGHT on effector T cells could contribute to the tumor regression observed in our experiments. In addition to LT-βR, LIGHT can bind to other two receptors, herpes virus entry mediator (HVEM) and decoy receptor 3/TR6 [21,35]. Several studies indicate that LIGHT can trigger apoptosis as well as cell activation depending on the expression of different receptors on the targeted cells [20,22]. Shaikh et al. showed that the constitutive expression of LIGHT on T cells led to inflammation and tissue destruction [36]. Tamada et al. showed that expression of LIGHT by transplanted tumors led to increased lymphocytic infiltrates, tumor necrosis and enhanced T cell cyotoxicity [37]. Similarly, Schrama et al. demonstrated that targeting LT-α3 to tumor resulted in tumor destruction via the formation of lymphoid-like structure in tumor sites [38]. It has been well documented that LT-βR signaling, and to a lessor extent, TNFR signaling is critical for the development and maturation of secondary lymphoid tissues [18,39]. One critical function of LT-βR is the activation of a chemokine-driven positive feedback loop required for the organization of lymphoid follicles [40]. Interestingly, a recent reported LT-βR signaling by LIGHT at tumor sites could lead to eradiation of well-established tumors via the recruitment of immune cells, including naïve T cells, and the formation of lymphoid-like structure inside tumors [41]. We hypothesized that one important function for LT-βR in our model is the activation of a similar chemokine-driven positive feedback loop by the adoptively transferred effector T cells that results in the recruitment of host innate cells, such as macrophages and dendritic cells, indirectly contributing to the tumor destruction process. Although it is conceivable that blocking with LT-βR might prevent the initial infiltration of adoptive effector T cells into the lungs, we did not observe a difference in the trafficking of CFSE-labeled effector T cells with or without LT-βR Fc treatment (data not shown). Thus, at least for our pulmonary metastases model, the effect of LT-βR blockage was unlikely due to the prevention of T cell trafficking. The fact that the therapeutic efficacy of wt effector T cells was not affected by LT-βR blockage is an additional argument against this possibility. The chemokines RANTES, MCP-1 and KC are induced in most inflammatory conditions and their expression correlates with the influx of macrophages into inflammatory sites [42-44]. In our in vitro experiments we detected the expression of RANTES, IP-10, KC and MCP-1 by D5 tumor cells after incubation with recombinant LT-α1β2. While growing tumors can likely counteract the immune system to insure their progression, it is of interest to note that the effector T cells may co-opt tumor cells themselves to contribute to their own demise. Further investigations into this paradox are warranted.

Together with other published data, our current study suggests that tumor-reactive T cells are capable of mediating tumor regression via a number of compensatory effector mechanisms. Recently, the clinical significance of tumor infiltrating lymphocytes was highlighted in multiple studies of human tumors, including colon cancer, ovarian cancer, and lymphoma [46-48]. Because not all possible effector molecules were examined, it will be of great interest to examine which particular effector mechanisms can be directly correlated to the patient's survival. Subsequently, strategies that induce these properties in T cells ex vivo could be applied to the adoptive immunotherapy of cancer, while alternatives that can induce these properties in vivo may serve as a useful adjunct for cancer vaccine strategies.

## Abbreviations

PKO, perforin knock out

GKO, IFN-γ knock out

PKO/GKO, perforin and IFN-γ double knock out

LKO, lymphotoxin knock out

LT-βR, lymphotoxin beta receptor

LT-α1β2, lymphotoxin α1β2 heterotrimer

LIHGT, homologous to lymphotoxin, exhibits inducible expression, and competes with HSV glycoprotein D for herpes virus entry mediator

TVDLN, tumor vaccine draining lymph node

D5, a poorly immunogenic clone of B16F10 melanoma

D5-G6, a D5 clone that producing murine GM-CSF

KC, a mouse homolog of human chemokine gro-alpha (CXCL1)

IP-10, γ-interferon-inducible protein

MCP-1, monocyte chemoattractant protein-1

Mig, monokine induced by γ-interferon

MIP-1, macrophage inflammatory protein

## Competing interests

The author(s) declare that they have no competing interests.

## Authors' contributions

BAF and H-MH conceived initial experiments to assess the role of LT-α as an anti-tumor effector mechanism. HW, CHP, BAF and H-MH designed and performed adoptive transfer experiments. Chemotaxis and molecular studies were designed and performed experiments by HW, NKE and H-MH. FAH and BAF was directly involved in drafting and revising the manuscript. HW, BAF, and H-MH were involved in data interpretation and the preparation and critical review of the manuscript.
